# Post-COVID-19 Fatigue: A Case of Infectious Hypothyroidism

**DOI:** 10.7759/cureus.14815

**Published:** 2021-05-03

**Authors:** Adrian Whiting, Jonathan Vincent M Reyes, Saad Ahmad, Joseph Lieber

**Affiliations:** 1 Internal Medicine, St. George's University School of Medicine, St. George's, GRD; 2 Internal Medicine, Icahn School of Medicine at Mount Sinai, Elmhurst Hospital Center, Elmhurst, USA; 3 Internal Medicine/Nephrology, Icahn School of Medicine at Mount Sinai, Elmhurst Hospital Center, Elmhurst, USA

**Keywords:** hypothyroidism, covid-19

## Abstract

Since the beginning of the coronavirus disease 2019 (COVID-19) global pandemic, an array of different clinical sequela and comorbid conditions have been discovered to be associated with COVID-19 infection. Of these sequela, subacute thyroiditis (SAT) causing hyperthyroidism has been prominent, more commonly affecting women. However, our case details a 49-year-old male patient with no history of thyroid disease showing signs and symptoms of hypothyroidism for six months after recovery from COVID-19 infection. His blood work was consistent with hypothyroidism, showing markedly elevated thyroid-stimulating hormone (TSH), suppressed T3 levels, and positive anti-thyroid peroxidase antibody titers. The patient was treated with Synthroid and showed quick clinical improvement in symptoms. This case demonstrates that COVID-19 infection can cause overt hypothyroidism in male patients, adding yet another clinical sequela of COVID-19 infection to our clinical repertoire from recently published case reports.

## Introduction

Clinical hypothyroidism is present in roughly 4.6% of US adults [1]. Common symptoms of hypothyroidism include dry skin, constipation, hypothermia, bradycardia, weakness, myalgia, and muscle cramps. Some patients may also present with pericardial effusion, diastolic hypertension, rhabdomyolysis, or even pseudohypertrophy of the muscles [2]. While many studies have shown the onset of subacute thyroiditis (SAT) after certain infections, including coronavirus disease 2019 (COVID-19), few studies have demonstrated the relationship between COVID-19 and overt hypothyroidism. Our objective is to detail the first known case of post-COVID-19 hypothyroidism in a 49-year-old male patient.

## Case presentation

A 49-year-old male with no significant past medical history was seen in the primary care clinic complaining of a six-month history of fatigue, unintentional 10-pound weight gain, constipation, dry skin, and myalgia. His fatigue was noticeable to the patient and severely affected his activities of daily living. He denied a personal or family history of hypothyroidism or any autoimmune disease. He denied any kind of medication use. He was diagnosed with COVID-19 in March 2020 and had an uncomplicated course and recovery. Of note, this was the first time this patient had experienced any symptoms like this before.

Vital signs were within normal limits (Table 1). The patient was not bradycardic. The physical exam was also within normal limits. There was no tenderness to palpation of the anterior neck and no palpably enlarged thyroid. No appreciable weight gain could be seen since his last clinic visit. 

**Table 1:** Patient's Vital Signs at Initial Visit

Vital Sign	Patient’s Value
Temperature	97.0F (36.1C)
Blood Pressure	134/69 mmHg
Heart Rate	96 beats per minute
Respiratory Rate	18 breaths per minute
Oxygen Saturation	97% on room air

Lab results were significant for elevated thyroid-stimulating hormone (TSH) of 74 and a detectable anti-thyroid peroxidase level of 626 (Table 2). TSH was repeated with similar elevation to 72. A severe acute respiratory syndrome coronavirus 2 (SARS-CoV-2) antibody test was reactive and revealed elevated titers to 137.7. The patient was initiated on Synthroid 50 mcg daily with improvement in symptoms and continues to follow up in the primary care clinic.

**Table 2:** Patient's Initial and Follow-Up Lab Values Anti-TPO: anti-thyroid peroxidase; SARS-CoV-2: severe acute respiratory syndrome coronavirus 2

Test	Reference Range	Patient’s Initial Visit	Patient’s Follow-Up Visit (6 Weeks Later)
Thyroid Stimulating Hormone (TSH)	0.27-4.20 mIU/L	74	22.75
Thyroglobulin (TG)	1.60-59.90 ng/mL	---	<0.2
Free T3/Free T4	Free T3: 1.80-4.60 pg/mL, Free T4: 0.93-1.70 ng/dL	---	3.12/0.72
Anti-TPO Antibodies	<34.9 IU/mL	626	---
SARS-CoV-2 Antibodies	<0.99	137.7 (Reactive)	---

## Discussion

SAT is a well-documented clinical sequela of COVID-19 infection [3]. However, several recent case studies describe what we commonly see: COVID-19 infection causing symptoms of hyperthyroidism, rather than hypothyroidism as published by Mehmood et al. [4]. This hyperthyroidism also more commonly affects women [5]. Our case is unique in that it is not only, to our knowledge, the first documented case of COVID-19 infection causing postinfectious hypothyroidism, but the first case of postinfectious hypothyroidism in a male patient. Additionally, the classical picture of a thyroid gland that is tender to palpation was also not observed in this patient [5]. 

The proposed mechanism for SAT in SARS-CoV-2 infection is via angiotensin-converting enzyme-2 (ACE-2) receptors present on the thyroid gland [6]. SARS-CoV-2 hijacks these receptors in order to enter the thyroid follicular cells. Infiltration of the thyroid gland through the ACE-2 receptors activates the cytokine storm, driven by interleukin-6 (IL-6) which launches an almost “autoimmune-like” attack against the thyroid gland. The immune-mediated response against thyroid follicular cells can cause one of two outcomes: it can destroy follicular cells, releasing preformed thyroid hormone into the systemic circulation, causing overt hyperthyroidism, which is what we see occurring in a majority of cases [3]. In a subset of cases, the cytokine storm can destroy follicular cells and the thyroid’s innate ability to produce T3/T4, causing a classical picture of Hashimoto’s thyroiditis leading to hypothyroidism: elevated TSH, low T3/T4, and the presence of anti-thyroid peroxidase antibodies, which is what we observed in this patient [6,7].

While widespread cases of postinfectious hypothyroidism have not been documented due to COVID-19 infection, clinically euthyroid patients hospitalized for COVID-19 developed transiently elevated TSH during hospitalization. In all cases of postinfectious SAT causing hyperthyroidism and isolated cases of elevated TSH in hospitalized patients, these were all transient phenomena that responded well to conservative treatment [8]. This patient was treated with guideline directed therapy for hypothyroidism and had complete resolution of his symptoms.

Another important consideration when assessing a patient for hypothyroidism is reversible causes: medications, a personal or family history of other autoimmune conditions, radiation exposure, or recent thyroid surgery. This patient did not take any medications, had no personal or family history of any autoimmune conditions, and denied any radiation exposure or thyroid surgeries. A thorough history is needed in order to rule out these sometimes overlooked causes of hypothyroidism before it can definitively be concluded that the hypothyroidism is secondary to an infectious etiology. As was the case with this patient, the only pertinent history he had was a recent history of COVID-19 infection with symptoms of hypothyroidism beginning almost immediately after he recovered from his COVID-19 infection. This temporal relationship between his COVID-19 infection and his hypothyroidism, in the absence of any other potentiating risk factors, helps us conclude that his hypothyroidism was most likely a sequela of his COVID-19 infection.

## Conclusions

This case will inform clinicians of another complication linked to the SARS-CoV-2 virus and add to an increasing number of vast pathologies associated with post-COVID-19 infection. As new information begins to emerge, it is important for clinicians to be aware of these evolving complications of the SARS-CoV-2 virus, so that they can investigate and address these issues earlier in order to lower morbidity and mortality rates, and improve patient care overall.

## References

[1] (2021). NIH: hypothyroidism (underactive thyroid). https://www.niddk.nih.gov/health-information/endocrine-diseases/hypothyroidism.

[2] Gurala D, Rajdev K, Acharya R, Idiculla PS, Habib S, Krzyzak M (2019). Rhabdomyolysis in a young patient due to hypothyroidism without any precipitating factor. Case Rep Endocrinol.

[3] Caron P (2020). Thyroid disorders and SARS-CoV-2 infection: from pathophysiological mechanism to patient management [Article in English, French]. Ann Endocrinol (Paris).

[4] Mehmood MA, Bapna M, Arshad M (2020). A case of post-COVID-19 subacute thyroiditis. Cureus.

[5] Ruggeri RM, Campennì A, Siracusa M, Frazzetto G, Gullo D (2021). Subacute thyroiditis in a patient infected with SARS-COV-2: an endocrine complication linked to the COVID-19 pandemic. Hormones.

[6] Pal R (2020). COVID-19, hypothalamo-pituitary-adrenal axis and clinical implications. Endocrine.

[7] Nalbandian A, Sehgal K, Gupta A (2021). Post-acute COVID-19 syndrome. Nat Med.

[8] Brancatella A, Ricci D, Cappellani D, Viola N, Sgrò D, Santini F, Latrofa F (2020). Is subacute thyroiditis an underestimated manifestation of SARS-CoV-2 infection? Insights from a case series. J Clin Endocrinol Metab.

